# Supplementary material for: Molecular conformation of polyelectrolytes inside Layer-by-Layer assembled films
Source: Nat Commun. 2023 Jul 10;14:4076. doi: 10.1038/s41467-023-39801-x (PMC10333193; doi:10.1038/s41467-023-39801-x)
Supplement: Supplementary file 1 — Supplementary Information [file 41467_2023_39801_MOESM1_ESM.pdf]

Supplementary Information: Molecular conformation of polyelectrolytes inside Layer-by-Layer assembled films

Philipp Gutfreund* and Giovanna Fragneto†
Institut Laue-Langevin, 71 avenue des Martyrs, 38042 Grenoble, France

Christophe Higy
Institut Laue-Langevin, 71 avenue des Martyrs, 38042 Grenoble, France and
Institut Charles Sadron, Université de Strasbourg, 67034 Strasbourg, France

Michel Tschopp, Olivier Félix, and Gero Decher
Institut Charles Sadron, Université de Strasbourg, 67034 Strasbourg, France

MORE DETAILS ON MATERIALS AND METHODS

Materials

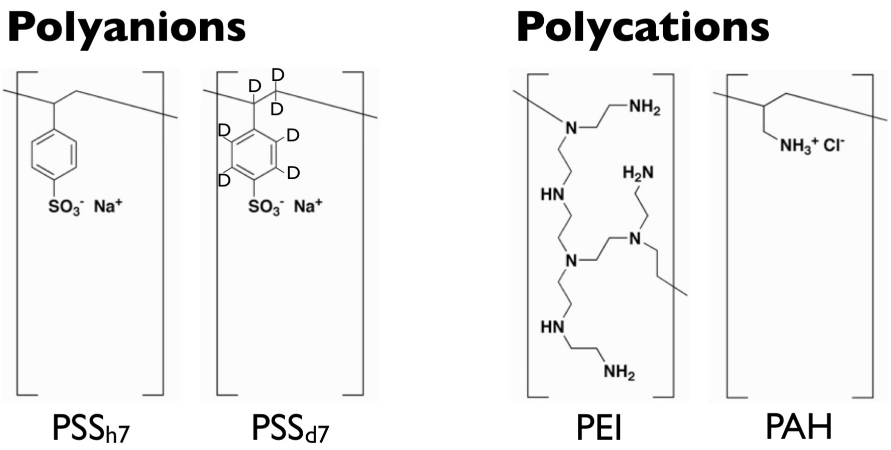

Supplementary Figure 1: Chemical structures of the polyelectrolytes used for the build-up of LbL films.

ADDITIONAL DATA

Specular Neutron Reflectometry

Preparation by spraying, 0.5M NaCl

In order to determine whether films prepared in the same conditions lead to the same structure and to study the reproducibility of the film build-up, we decided to use the "Global Fit" option of Motofit[1]. O. Félix *et al.* [2] already investigated this idea by determining the structural parameter values from two films either fully deuterated or fully protonated. They observed that it is possible to use structural values of some other films prepared in the same

* gutfreund@ill.eu
† Current address: European Spallation Source ERIC, P.O. Box 176, 22100 Lund, Sweden

conditions, but not for all them. In our case, we went further by using the "Global Fit" process, which allows to fit several reflectivity curves at the same time, with the possibility to link the parameters of the different films together, so that one value of the parameters is calculated for all the films. For example, it is possible to determine one value of the thickness per layer pair for all the films. Then, by comparing the global fit results with the individual fits, it is possible to determine if the parameters are the same for all the films or not.

For this study, we analysed the specular reflectivity curves measured at LLB in 2007[2] for the sixteen multilayer films prepared by spraying in the same conditions :

- Sample A : $(PSS_{d7}\text{-}PAH)_{10}$
- Sample B : $(PSS_{h7}\text{-}PAH)_{10}$
- Sample C : $[(PSS_{h7}\text{-}PAH)_3/(PSS_{d7}\text{-}PAH)]_6/(PSS_{h7}\text{-}PAH)_3$
- Sample D : $[(PSS_{h7}\text{-}PAH)_5/(PSS_{d7}\text{-}PAH)]_6/(PSS_{h7}\text{-}PAH)_5$
- Sample E : $[(PSS_{h7}\text{-}PAH)_4/(PSS_{d7}\text{-}PAH)]_8/(PSS_{h7}\text{-}PAH)_4$
- Sample G : $[(PSS_{h7}\text{-}PAH)_4/(PSS_{d7}\text{-}PAH)_2]_6/(PSS_{h7}\text{-}PAH)_4$
- Sample H : $[(PSS_{h7}\text{-}PAH)_3/(PSS_{d7}\text{-}PAH)_3]_6/(PSS_{h7}\text{-}PAH)_3$
- Sample I : $[(PSS_{h7}\text{-}PAH)_2/(PSS_{d7}\text{-}PAH)_4]_6/(PSS_{h7}\text{-}PAH)_2$
- Sample J : $[(PSS_{h7}\text{-}PAH)/(PSS_{d7}\text{-}PAH)_5]_6/(PSS_{h7}\text{-}PAH)$
- Sample K : $[(PSS_{h7}\text{-}PAH)_5/(PSS_{h7}\text{-}PAH)]/[(PSS_{h7}\text{-}PAH)_5/(PSS_{d7}\text{-}PAH)]_4/$ $(PSS_{h7}\text{-}PAH)_5$ or $(PSS_{h7}\text{-}PAH)_{11}/[(PSS_{d7}\text{-}PAH)/(PSS_{h7}\text{-}PAH)_5]_4$
- Sample L : $[(PSS_{h7}\text{-}PAH)_5/(PSS_{d7}\text{-}PAH)]/[(PSS_{h7}\text{-}PAH)_5/(PSS_{h7}\text{-}PAH)]_4/$ $(PSS_{h7}\text{-}PAH)_5$ or $(PSS_{h7}\text{-}PAH)_5/(PSS_{d7}\text{-}PAH)/(PSS_{h7}\text{-}PAH)_{29}$
- Sample M : $[(PSS_{h7}\text{-}PAH)_5/(PSS_{d7}\text{-}PAH)]_2/[(PSS_{h7}\text{-}PAH)_5/(PSS_{h7}\text{-}PAH)]/$ $[(PSS_{h7}\text{-}PAH)_5/(PSS_{d7}\text{-}PAH)]_2/(PSS_{h7}\text{-}PAH)_5$ or $[(PSS_{h7}\text{-}PAH)_5/(PSS_{d7}\text{-}PAH)]_2/(PSS_{h7}\text{-}PAH)_{11}/[(PSS_{d7}\text{-}PAH)/(PSS_{h7}\text{-}PAH)_5]_2$
- Sample N : $[(PSS_{h7}\text{-}PAH)_5/(PSS_{h7}\text{-}PAH)]_2/[(PSS_{h7}\text{-}PAH)_5/(PSS_{d7}\text{-}PAH)]/$ $[(PSS_{h7}\text{-}PAH)_5/(PSS_{h7}\text{-}PAH)]_2/(PSS_{h7}\text{-}PAH)_5$ or $(PSS_{h7}\text{-}PAH)_{17}/(PSS_{d7}\text{-}PAH)/(PSS_{h7}\text{-}PAH)_{17}$
- Sample O : $[(PSS_{h7}\text{-}PAH)_5/(PSS_{d7}\text{-}PAH)]_4/[(PSS_{h7}\text{-}PAH)_5/(PSS_{h7}\text{-}PAH)]/$ $(PSS_{h7}\text{-}PAH)_5$ or $[(PSS_{h7}\text{-}PAH)_5/(PSS_{d7}\text{-}PAH)]_4/(PSS_{h7}\text{-}PAH)_{11}$
- Sample P : $[(PSS_{h7}\text{-}PAH)_5/(PSS_{h7}\text{-}PAH)]_4/[(PSS_{h7}\text{-}PAH)_5/(PSS_{d7}\text{-}PAH)]/$ $(PSS_{h7}\text{-}PAH)_5$ or $(PSS_{h7}\text{-}PAH)_{29}/(PSS_{d7}\text{-}PAH)/(PSS_{h7}\text{-}PAH)_5$
- Sample Q : $[(PSS_{h7}\text{-}PAH)_6/(PSS_{d7}\text{-}PAH)]_8/(PSS_{h7}\text{-}PAH)_6$

Preparation by dipping 2M NaCl

One example of a dipped film with the following structure is shown: $[(PSS_{h7}\text{-}PAH)_3/(PSS_{d7}\text{-}PAH)]_6$. The specular reflectivity curve measured on N-REX+ with the corresponding fit are shown in Supplementary Figure 4, as well as the SLD profile. The structural parameter values of the best fit are summarized in Supplementary Table I.

	d [Å]	σ [Å]	SLD_d $[10^{-6}Å^{-2}]$	SLD_h $[10^{-6}Å^{-2}]$
Top layer	10 ± 9	18 ± 9	8 ± 4	X
Bulk layers	52 ± 2	17 ± 10	3.3 ± 0.3	1 ± 0.3
Bottom layer	52 ± 6	18 ± 15	X	1 ± 0.4

Supplementary Table I: Fitting parameters for the sample from Supplementary Fig.4

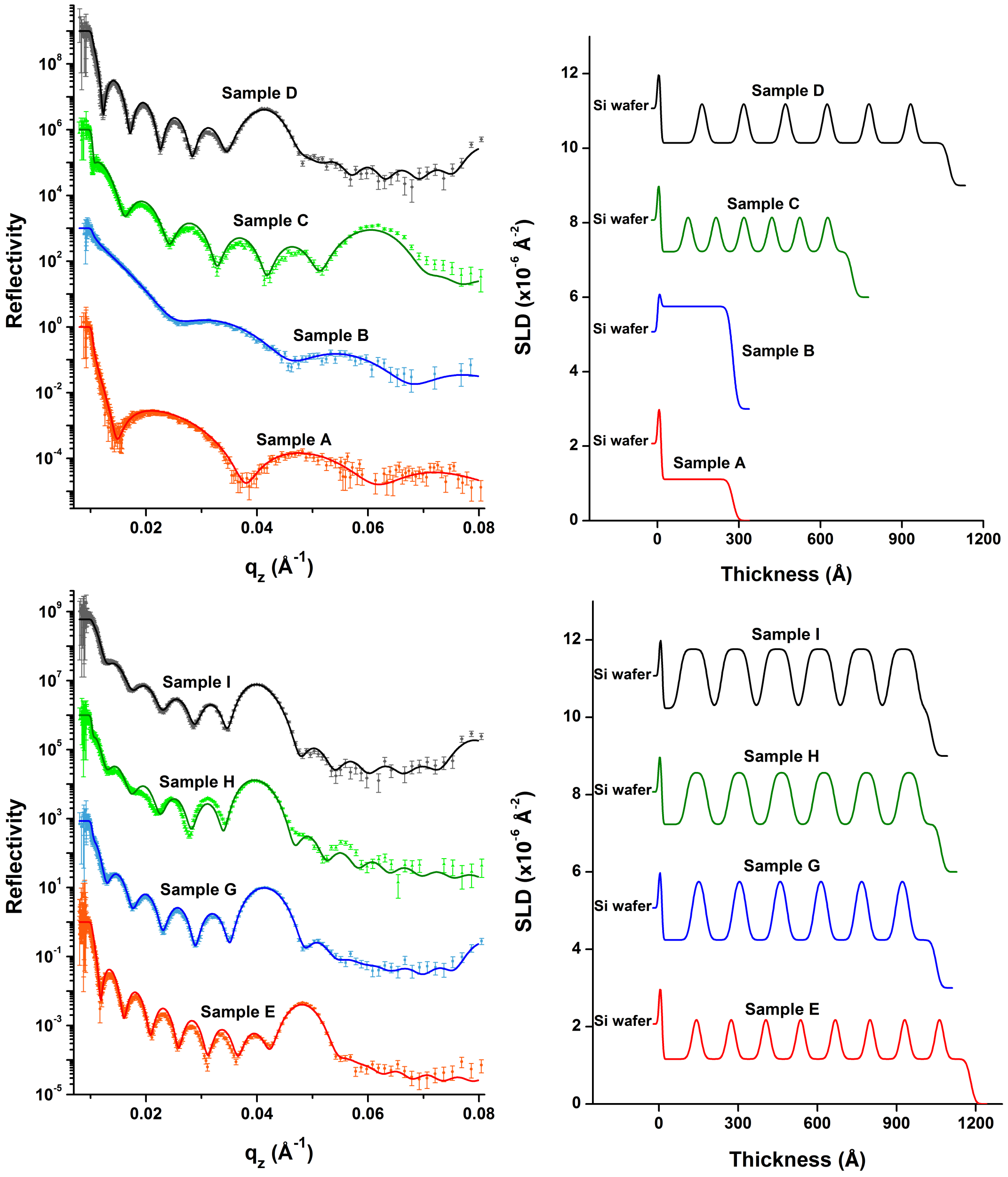

Supplementary Figure 2: On the left, the experimental specular reflectivity curves (points) measured at LLB in 2007 and the corresponding theoretical models (line). On the right, the SLD profiles extracted from the fits. The curves are shifted for clarity. Error bars indicate the statistical counting error.

	d_{PAH} [Å]	d_{PSS} [Å]	σ_{PAH} [Å]	σ_{PSS} [Å]	SLD$_{\mathrm{PAH}}$ [10^{-6}Å^{-2}]	SLD$_{\mathrm{PSS}}$ [10^{-6}Å^{-2}]
Bulk+top layers	17 ± 3	35 ± 3	25.5 ± 1	25.5 ± 1	0.1 ± 0.3	2.4 ± 0.2
Bottom layer	17 ± 3	35 ± 3	25 ± 10	35 ± 5	0.1 ± 0.3	2.3 ± 0.2

Supplementary Table II: Fitting parameters for the sample from Fig. 1a in the main article.

In addition we report in Supplementary Tab. II the fitting parameters for the curve shown in Fig. 1a of the main article. The multilayer structure is [(PSS$_{70\%d7}$-PAH)$_{73}$ prepared by dipping.

Finally, we report a specular reflectivity curve of a sample stored for 15 years at ambient conditions with the following structure: [(PSS$_{h7}$-PAH)$_2$/(PSS$_{d7}$-PAH)]$_8$. In Supplementary Fig. 5 it can be seen that the Bragg-peak related to

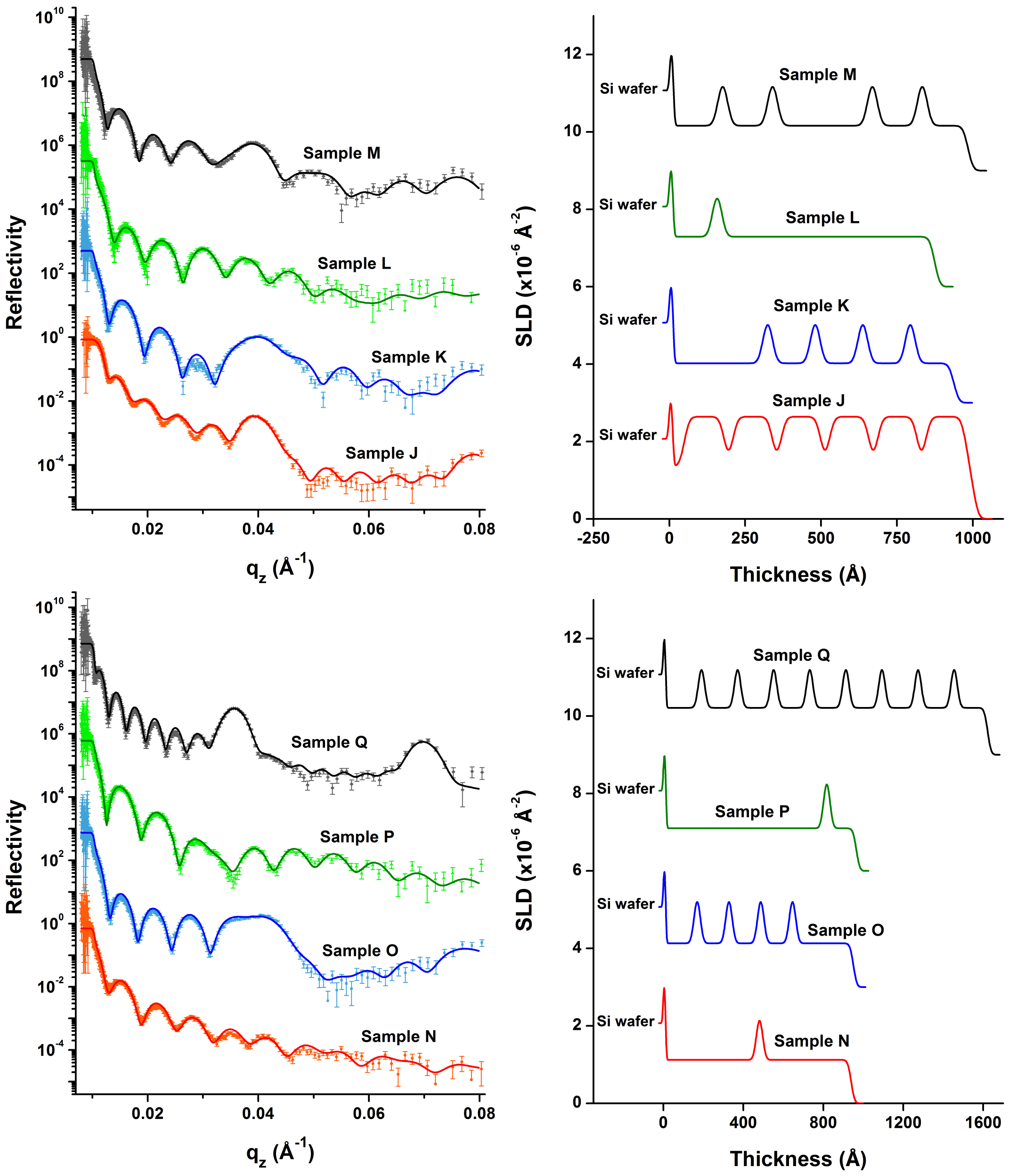

Supplementary Figure 3: On the left, the experimental specular reflectivity curves (points) measured at LLB in 2007 and the corresponding theoretical models (line). On the right, the SLD profiles extracted from the fits. The curves are shifted for clarity. Error bars indicate the statistical counting error.

the inserted deuterated layers is still present, testifying the effective "freezing" of non-equilibrium structures in these solid-like films.

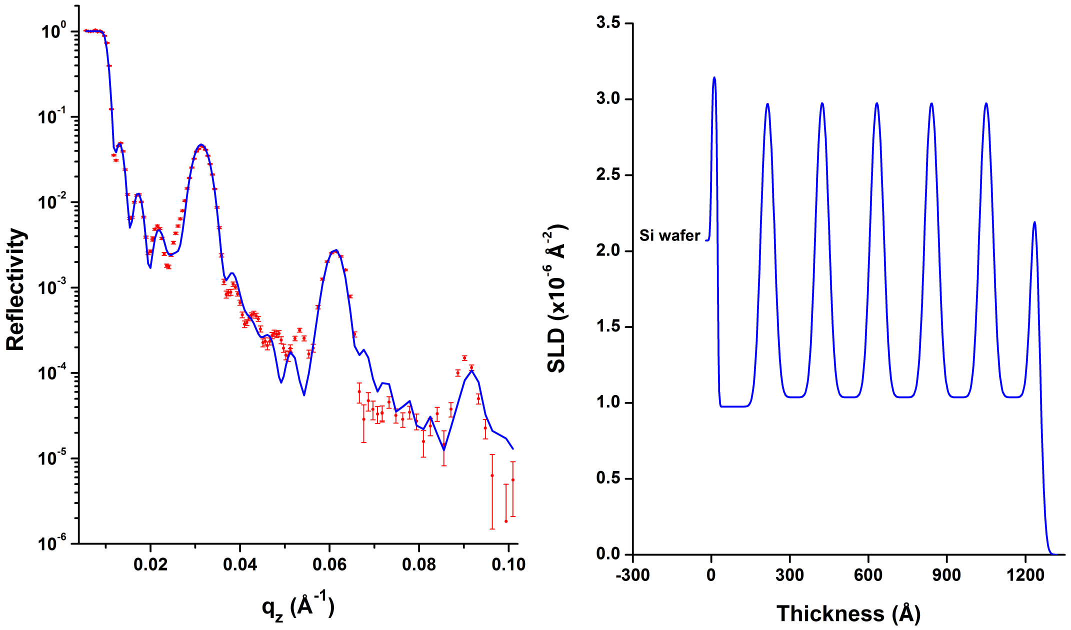

Supplementary Figure 4: On the left, the experimental specular reflectivity curve (points) and the corresponding theoretical model (line). On the right, the SLD profile extracted from the fit. Error bars indicate the statistical counting error.

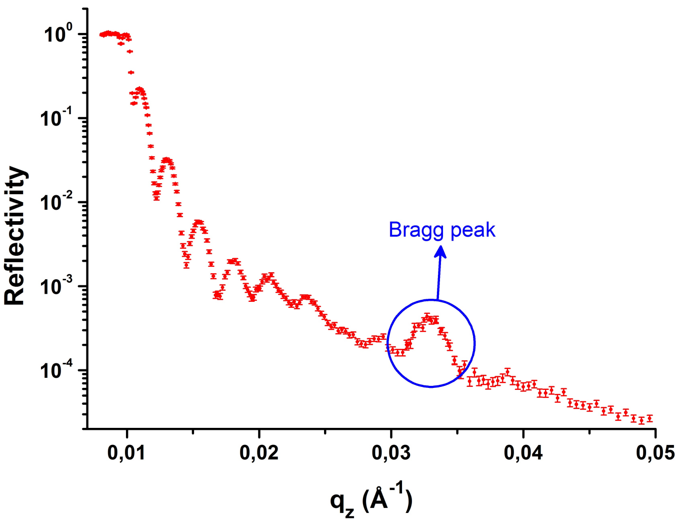

Supplementary Figure 5: Experimental specular reflectivity curve of a sample stored for 15 years at ambient conditions. Error bars indicate the statistical counting error.

SANS

Isotope effects in SANS

The theoretical scattering cross section under the assumption of slight incompatibility of deuterated and protonated chains is given by the Random Phase Approximation [3]:

$$\left(\frac{\mathrm{d}\sigma}{\mathrm{d}\Omega}\right)^{-1} = (N_{bD} - N_{bH})^{-2} v_P^{-1} * \left(\frac{1}{\Phi_D n_P F(\vec{q})} + \frac{1}{(1-\Phi_D) n_P F(\vec{q})} + v_e\right). \tag{1}$$

Here, v_e is the excluded volume parameter or second virial coefficient in case of deviations from the ideal Gaussian chain. In essence, if slight isotope effects are taken into account the total cross-section of the single chain form factor keeps its original shape, but the toal cross-section at zero q would be overestimated by a simple homogeneous Fit (eq. 2). In the here studied case the molecular weight of 80800 g/mol is relatively low and thus isotope effects are unlikely to play a significant role.

Estimation of free radius of gyration of PSS in complexes

M. Z. Markarian *et al.* have studied the size of PSS chains in bulk complexes of polyelectrolytes [4]. The radius of gyration of PSS chains in a PSS/(diallyldimethylammonium chloride) (PDADMAC) complex was determined by SANS, for two different molecular weights of the PSS (M_w = 14000 g/mol and M_w = 104000 g/mol) and for concentration of salt (sodium chloride, NaCl) in the solutions from 0.1 M to 1.5 M. The conformations were determined to be more or less spherical, and the sizes were 25 Å to 27 Å for the low PSS molecular weight and 105 Å to 110 Å for the high PSS molecular weight.

Taking the radius of gyration of the 104000 g/mol PSS in a 2 M NaCl PDADMA complex as a starting point and scaling to our molecular weight of 80,800 g/mol we can estimate the bulk radius of gyration of the PSS in the complex being around 90 Å in our case.

Details of the SANS modeling

In analogy to previous SANS studies on polyelectrolyte/salt complexes[5] we opted for a model consisting of three distinct contributions: The scattering from fractals at low q-values, the scattering from single polymer chains at larger q-values and a flat background due to incoherent scattering at the largest q values.

This was accomplished either by using the fitting program SASFit [6] or by using a hand-written code as explained later. In SASFit the low-q region was modeled as a mass fractal and the best fit for the dipped sample was obtained with an exponential cut-off correlation length of $\xi = 850$ Å and a gauge of $r_0 = 81$ Å and a fractal dimension of $D = 2$ pointing towards a random walk structured agglomerate. The sprayed sample showed stronger fractal scattering and was best fitted with a correlation length of 1230 Å, a gauge of 46 Å and a fractal dimension of $D = 2.95$, reveling more clustered and larger agglomerates. We note here that different samples prepared the same way showed different scattering in this low q region, pointing towards a non-reproducible character of these fractal structures even among different samples prepared the same way. The generally stronger fractal scattering of the sprayed samples compared to the dipped ones was always observed, though. However, whatever the exact fractal parameters used to model these large structures the fitting results for the smaller sized structures were only little influenced, at least for the dipped samples. In SASFit we used two approaches to fit the higher q-region of the SANS curves: a) Either a worm-like chain model (e.g. Kholodenko worm) was used to fit this entire range, which allowed to fit the chain contour length, the Kuhn length (b) and the scattering contrast, or, b) a sum of two contributions with the smaller size contribution responsible for the high-q region showing a power law of -1, modeled as a freely joined chain of (infinitely thin) rods of a fitted Kuhn length and arbitrarily fitted scattering strength and a second contribution for intermediate sizes, modeled as a Gaussian distribution of (point-like) monomers resulting in the well-known Debye-function if Fourier-transformed into q-space. In both of these approaches the best fit was obtained assuming a Kuhn length of 10 Å for all samples, recovering the known Kuhn length for PSS [7]. The single-chain radius of gyration in direction parallel to the surface for the dipped sample turned out to be $R_g^{||} = 140 \pm 20$ Å for the worm-like chain model and $R_g^{||} = 180^{+20}_{-10}$ Å for the Debye fit, the latter being well in accord with the model-free Guinier analysis presented above. The absolute intensity of this contribution turned out to be $20 \pm 3\,\mathrm{cm}^{-1}$ in the worm-like chain model and $16 \pm 4\,\mathrm{cm}^{-1}$ for the Debye fit, both being within the theoretical values extracted from the neutron scattering contrast between the deuterated and protonated PSS chains, their concentration and molecular weight as explained in the Methods section. For the sprayed sample the wormlike chain model revealed the same value for $R_g^{||}$ (140 Å) and scattering intensity ($20\,\mathrm{cm}^{-1}$), whereas the Debye model fitted $R_g^{||} = 220$ Å with a scattering contrast of $18\,\mathrm{cm}^{-1}$ for the sprayed sample.

Alternatively, we used a home-written code to fit the SANS curves using the approach presented in Refs. [7, 8], often used for PE solutions which assumes infinitely long worm-like chains resulting in the asymptotic behaviour calculated by de Cloizeaux. Here we also added a constant background and the fractal part as in SASFit. The exact used function is presented in the Methods section. Using this approach the fitting results tuned out to be rather ambiguous, though. The best fit for the fractal scattering was achieved using $D = 2.5$, $r_0 = 130$ Å and $\xi = 380$ Å for the dipped sample and

$D = 2.93$, $r_0 = 49$ Å and $\xi = 1300$ Å for the sprayed sample rather consistent with the SASFit analysis. The effective monomer length turned out to be significantly larger than for bulk PSS ($a \sim 2$ Å) and could be fitted to 4 Å for the dipped sample and 5 Å for the sprayed one. The persistence length turned out to be 87 Å for the dipped sample, which is also larger than values reported in pure PSS solutions (36 Å $< l_p <$ 77 Å), reflecting the qualitative result obtained from the power law change-over momentum transfer discussed above. For the sprayed samples $l_p = 41$ Å gave the best fit, this value, however, has to be taken with care as accompanied with a large uncertainty due to the strong small q scattering. Similarly for the radius of gyration, the analysis of the dipped sample revealed a value of 230±60 Å, while for the sprayed one $R_g^{\|} = 150$ Å showed the best fit (both with a scattering contrast around 25 cm^{-1}). But again, the radius of gyration came in with a very large fitting error and a value of $R_g^{\|} = 180$ Å fitted both of the data equally well. In general, due to the strong low q scattering of the sprayed samples (and spin-coated samples, not shown), the single chain parameters $R_g^{\|}$ and l_p are very difficult to extract as the fractal scattering characterised by a steep q-power law directly turns into the chain stiffness power law ($\sim q^{-1}$) overwhelming the scattering from the entire chain's form ($\sim q^{-2}$). Therefore these parameters extracted from sprayed and spin-coated samples cannot be used and will not be discussed further, only the effective monomer size (a) or Kuhn length (b) could be extracted from these samples.

GISANS

The experimental curves for the three wavelengths falling onto a master curve are shown in Supplementary Figure 6.

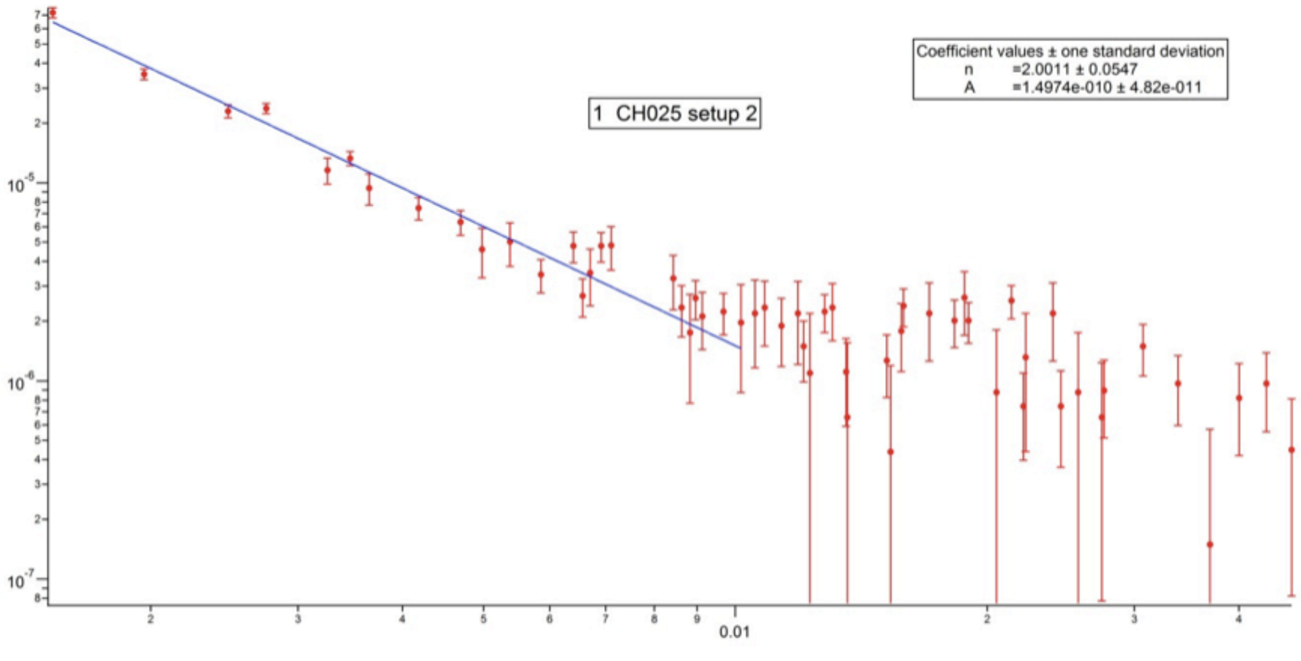

Supplementary Figure 6: Out-of-plane scans (GISANS Intensity vs q_y [Å^{-1}]) of the multilayer film of 53 bi-layers containing 50% deuterated PSS. The experimental data (points) for average wavelengths of 4 Å, 5 Å and 7 Å are shown. The line is a fit to the low q data reveling a power law with a slope of -2. Error bars indicate the statistical counting error.

The Guinier analysis of the same GISANS data at 11Å wavelength is shown in Supplementary Fig. 7.

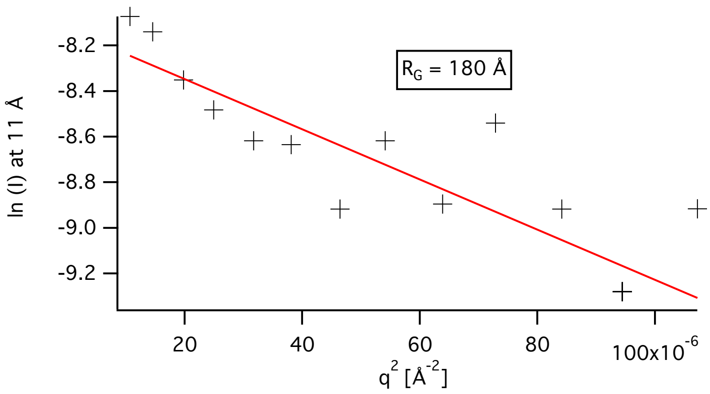

Supplementary Figure 7: Guinier plot of the GISANS intensity at 11Å of a dipped sample (same as in Supplementary Fig. 6). The slope is fitted by the solid line indicating a radius of gyration of 180 Å.

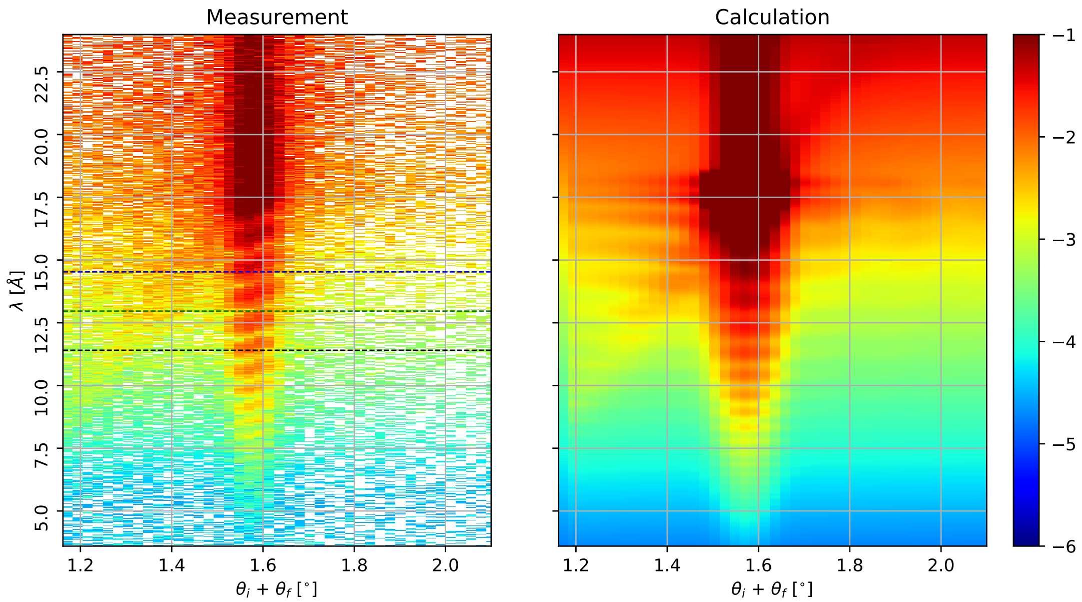

Supplementary Figure 8: OSS map of a dipped sample (same as in Fig. 2 of the main article in the experimental space, wavelength vs. reflection angle (reflected intensity on logarithmic color scale). Left: Experimental data, Right: Simulation.

OSS

---

[1] Nelson, A. Co-refinement of multiple-contrast neutron/x-ray reflectivity data using motofit. *Journal of Applied Crystallography* **39**, 273–276 (2006).

[2] Félix, O., Zheng, Z., Cousin, F. & Decher, G. Are sprayed lbl-films stratified ? a first nanostructure of spray-assembled multilayers by neutron reflectometry. *C. R. Chimie* **12**, 225–234 (2009).

[3] de Gennes, P. G. *Scaling Concepts in Polymer Physics* (Cornell University Press, 1979).

[4] Markarian, M. Z., Hariri, H. H., Reisch, A., Urban, V. S. & Schlenoff, J. B. A small-angle neutron scattering study of the equilibrium conformation of polyelectrolytes in stoichiometric saloplastic polyelectrolyte complexes. *Macromolecules* **45**, 1016–1024 (2012).

[5] Murphy, R. J. *et al.* Scattering studies on poly(3,4-ethylenedioxythiophene)-polystyrenesulfonate in the presence of ionic liquids. *Macromolecules* **48**, 8989–8997 (2015). URL `https://doi.org/10.1021/acs.macromol.5b02320`. https://doi.org/10.1021/acs.macromol.5b02320.

[6] Breßler, I., Kohlbrecher, J. & Thünemann, A. F. Sasfit: a tool for small-angle scattering data analysis using a library of analytical expressions. *Journal of applied crystallography* **48**, 1587–1598 (2015).

[7] Combet, J. Polyelectrolytes and small angle scattering. In *EPJ Web of Conferences*, vol. 188, 03001 (EDP Sciences, 2018).

[8] Dubois, E. & Boué, F. Conformation of poly(styrenesulfonate) polyions in the presence of multivalent ions: Small-angle neutron scattering experiments. *Macromolecules* **34**, 3684–3697 (2001).
